# Neuroprotective and Anti-Inflammatory Roles of the Phosphatase and Tensin Homolog Deleted on Chromosome Ten (PTEN) Inhibition in a Mouse Model of Temporal Lobe Epilepsy

**DOI:** 10.1371/journal.pone.0114554

**Published:** 2014-12-12

**Authors:** Valentina Grande, Giusi Manassero, Alessandro Vercelli

**Affiliations:** 1 Neuroscience Institute Cavalieri Ottolenghi, Department of Neuroscience, University of Torino, Orbassano, Torino, Italy; 2 Department of Internal Medicine, University of Genova, Genova, Italy; Univ. Kentucky, United States of America

## Abstract

Excitotoxic damage represents the major mechanism leading to cell death in many human neurodegenerative diseases such as ischemia, trauma and epilepsy. Caused by an excess of glutamate that acts on metabotropic and ionotropic excitatory receptors, excitotoxicity activates several death signaling pathways leading to an extensive neuronal loss and a consequent strong activation of astrogliosis. Currently, the search for a neuroprotective strategy is aimed to identify the level in the signaling pathways to block excitotoxicity avoiding the loss of important physiological functions and side effects. To this aim, PTEN can be considered an ideal candidate: downstream the excitatory receptors activated in excitotoxicity (whose inhibition was shown to be not clinically viable), it is involved in neuronal damage and in the first stage of the reactive astrogliosis *in vivo*. In this study, we demonstrated the involvement of PTEN in excitotoxicity through its pharmacological inhibition by dipotassium bisperoxo (picolinato) oxovanadate [bpv(pic)] in a model of temporal lobe epilepsy, obtained by intraperitoneal injection of kainate in 2-month-old C57BL/6J male mice. We have demonstrated that inhibition of PTEN by bpv(pic) rescues neuronal death and decreases the reactive astrogliosis in the CA3 area of the hippocampus caused by systemic administration of kainate. Moreover, the neurotoxin administration increases significantly the scanty presence of mitochondrial PTEN that is significantly decreased by the administration of the inhibitor 6 hr after the injection of kainate, suggesting a role of PTEN in mitochondrial apoptosis. Taken together, our results confirm the key role played by PTEN in the excitotoxic damage and the strong anti-inflammatory and neuroprotective potential of its inhibition.

## Introduction

The phosphatase and tensin homolog deleted on chromosome ten (PTEN) was initially studied extensively for its fundamental role in tumorigenicity: therefore, the multiple functions performed by PTEN in Central Nervous System (CNS) in both physiological and pathological conditions were initially underestimated and became the focus of several studies only recently. In the CNS, PTEN plays a fundamental role in development [1], [2], [3], synaptogenesis and synaptic plasticity [4], [5] and in neuronal death [6].

PTEN gene encodes for a phosphatase specific for both protein and lipid substrates [7], [8]. Both these activities can be involved in the regulation of neuronal death [6], [9]. As a lipid phosphatase, PTEN directly antagonizes the PI3K/AKT pathway which regulates growth, proliferation, survival, apoptosis and cell migration as well as the development and maintenance of the nervous system [10], [11]. Cultured hippocampal neurons, in which PTEN activity was decreased by overexpression of a dominant-negative mutant form of PTEN, showed an increase in the level of p-Akt (the active form of Akt) and, similarly to the neurons in mice with PTEN haploinsufficiency (PTEN^+/−^ mice), exhibited increased resistance to death induced by excitotoxicity [12]. As a protein phosphatase, PTEN regulates the function and surface expression of N-methyl-D-aspartate receptors (NMDARs), a key subtype of excitatory glutamate receptor known to mediate excitotoxicity-induced neuronal death [9]. However, despite the fundamental proapoptotic role played by the lipid and protein phosphatase activities, the regulation of neuronal death by PTEN appears much more complex. In fact it has been demonstrated that a death stimulus may also result in a subcellular redistribution of PTEN that, in normal conditions localized in the cytosol, translocates into mitochondria in cultured hippocampal cells treated with staurosporine (a relatively nonselective protein kinase inhibitor that can induce apoptosis in a broad spectrum of cells), suggesting the involvement of PTEN in mitochondria-dependent apoptosis [13]. Moreover, elevated levels of mRNA and PTEN protein have been shown in human Alzheimer's Disease (AD), where they contribute to the AD neurodegeneration [14], whereas other studies showed the link between PTEN and stress activated signaling pathways such as c-Jun N-terminal kinase (JNK) pathway [15]. Nevertheless, in spite of all these pieces of evidence, the role of PTEN in regulating neuronal death is still far from being fully understood.

To this aim, in our study we examined the effect of the pharmacological inhibition of PTEN in a model of excitotoxic neuronal death induced by intraperitoneal injection of kainic acid in mice.

The excitotoxic damage is considered the major mechanism underlying neuronal death in many human disease states such as ischemia, trauma and epilepsy. The term indicates an excess of glutamate that, acting on metabotropic and ionotropic excitatory receptors, causes cell death [16], [17]. As the glutamate, kainic acid (KA) is able to trigger excitotoxic neuronal death [18]. In rodents, systemic or intracerebral injection of KA, activating the kainate subtype of ionotropic glutamate receptors, results in sustained epileptic activity in the hippocampus, followed by a selective pattern of neuropathology characterized by a severe neuronal loss and by glial cell activation and similar to the human temporal lobe epilepsy [19], [20]. Treatment with antagonists of excitatory amino acids, the most common strategy to prevent excitotoxicity, has not proved clinically viable [21]; therefore, new neuroprotective strategies are focused downstream the excitatory amino acid receptors in order to target molecules in signaling pathways controlling neuronal death. In this context, PTEN can be considered as an ideal candidate for its involvement in neuronal damage and, probably, in reactive astrogliosis [22].

Here we show that PTEN inhibition by intraperitoneal administration of dipotassium bisperoxo (picolinato) oxovanadate [bpv(pic)] is able to partially prevent the massive neuronal loss and the reactive astrogliosis in the CA3 area of the hippocampus after systemic injection of kainic acid in C57BL/6J mice. Moreover, in animals treated with bpv(pic) the increased PTEN presence in mitochondria induced by kainic acid seems to be reduced 6 hours after the excitotoxin application.

## Materials and Methods

### Ethics statement

All experimental procedures on live animals were performed according to the European Communities' Council Directive of 24 November 1986 (86/609/EEC) and University of Torino's institutional guidelines for animal welfare (DL 116/92), authorization 17/2010-B (June 30, 2010) by Italian Ministry of Health; efforts were made to minimize the number of animals used and their suffering. The project was approved by the bioethic committee of the University of Torino (http://www.unito.it/unitoWAR/page/istituzionale/ricerca1/Ricerca_comitato1).

### Experimental animals

2-month-old C57BL/6J male mice (weight 20 g) were purchased from Janvier, St Berthevin Cedex, France. Animals had free access to food and water.

Mice were divided in 7 experimental groups (n = 4 for each group). Controls were injected with three intraperitoneal injections of 0.9% saline. Three groups of mice were treated with kainate and with two injections of 0.9% saline 30 minutes before and 90 min after stimulation with kainate and sacrificed at different time points after kainate injection (3, 6 or 12 h). Three groups received intraperitoneally kainate injection and two injections of bpv(pic) 30 minutes before and 90 minutes after the kainate stimulation and were sacrificed at different time points after kainate injection (3, 6 or 12 h).

Other three experimental groups were used for Nissl-staining analysis and for immunohistochemical analysis: (i) kainate-treated, 1 day survival (n = 3) intraperitoneally injected with kainate and with two injections of 0.9% saline 30 minutes before and 90 minutes after stimulation with kainate; (ii) kainate/bpv(pic)-treated, 1 day survival (n = 3) intraperitoneally injected with kainate and with two injections of bpv(pic) 30 minutes before and 90 min after stimulation with kainate; (iii) control animals, 1 day survival (n = 3) intraperitoneally injected with three injections of 0.9% saline at the same time points of the kainate and kainate/bpv(pic) animals.

### Induction of epileptic seizures and bpv(pic) administration

We induced epileptic seizures by intraperitoneal (i.p.) injection of 25 mg/kg kainate (Tocris Bioscience, Bristol, UK). Within one hour after administration of kainate, mice showed the first symptoms (immobility, facial myoclonus and head nodding), and only animals that reached the fifth stage of the Racine scale [19], [23] were included in the study: stage 0, no seizures; stage 2, head nodding; stage 3, forelimb clonus; stage 4, rearing in addition to severe forelimb clonus; stage 5, rearing and falling in addition to severe forelimb clonus. To minimize suffering and prevent mortality, 1 h following the onset of seizure, a single rectal administration of 8 mg/kg diazepam (Valium; Roche, Monza, Italy) blocked epileptic seizures within 30 min of administration. ALX-270-205-M005 bpV(pic) PTP inhibitor (2 mg/kg, Vinci-Biochem, Vinci, Italy), the PTEN inhibitor, was injected 30 minutes before and 90 minutes after stimulation with kainate. The dose of PTEN inhibitor (2 mg/kg) was chosen on the basis of previous studies by Sury and co-workers [24].

### Tissue preparation

Animals were killed with an overdose of anesthetic (i.p. chloral hydrate) and perfused through the left ventricle with washing phosphate buffer (0.1 M, pH 7.4), followed by 4% paraformaldehyde in phosphate buffer. Brains were removed from the skull and postfixed in the same fixative for 4 h. The tissue was cryoprotected by immersion in buffered 30% sucrose overnight, embedded, and frozen in cryostat medium (Bio-Optica, Milan, Italy). The brains were cut into coronal, 20 µm thick, free-floating sections, and stored in a cryoprotective solution at −20°C until being used for Nissl-staining and for immunohistochemical analysis.

Other animals were decapitated in deep anaesthesia. Just cut the heads of the mice, the brains were dissected and the hippocampus (both sides) was quickly removed, frozen and stored at -80°C until being used for Western Blotting analysis.

### Total protein isolation from mouse hippocampi

Mouse hippocampi (both sides, n = 3) were collected and stored at −80°C immediately after dissection from the brain. They were homogenized in RIPA lysis buffer supplemented with proteases (1× complete protease inhibitor cocktail, CPIC, Roche) and phosphatases (1× 4-nitrophenyl phosphate 4-NPP, Roche) inhibitors. Soluble fractions were prepared by centrifuging the total homogenates at 13,000 rcf for 10 min; the supernatant was collected and used for Western blot analysis. The total protein content was measured with the Bio-Rad Protein Assay kit (Bio-Rad) and loaded on a pre-cast mini-gel system with 4-12% Bis-Tris gradient to evaluate the phosphorylation status of Akt.

### Mitochondria and cytoplasm protein isolation

All subsequent steps were conducted at 4°C. Proteins from both the cytosolic and mitochondrial fractions were extracted according to the multiple centrifugation method described elsewhere [25] which was slightly modified. Briefly, hippocampal tissue was washed twice with ice-cold PBS and gently dounced 10 times in ice-cold isolation medium (250 mM Mannitol, 20 mM Tris, 1 mM EGTA, 1 mM EDTA and 0.3% w/v BSA plus 1% phosphatase inhibitor and protease inhibitor). The homogenates were stored on ice for 20 min and then centrifuged at 450 g for 10 min. The supernatants were transferred into centrifugation tubes. The subsequent centrifugation (22,000 g for 10 min) generated the cytosolic fraction and the mitochondrial pellet. The pellet was washed twice with isolation medium and then re-suspended in a denaturing lysis buffer (250 mM NaCl, Tris 20 mM, EDTA 3mM, EGTA 3 mM, Nonidet 0.5%, 1% phosphatase inhibitor and protease inhibitor) and incubated on ice for 30 min. Thereafter, the mitochondrial suspension was briefly sonicated and centrifuged to remove insoluble material (16,000 g for 15 min). Supernatants were normalized for cytosolic and mitochondrial proteins content (Bio-Rad Protein Assay kit; Bio-Rad) and stored at −80°C.

### Quantitative analysis

Neuronal cell counts were performed in CA3 area, in Nissl-staining coronal sections corresponding to plates 41, 44, 47 of the Paxinos and Franklin atlas [26], representing three different levels of the hippocampus, from rostral to caudal side, in order to consider 3 sections/mouse.

Neurons were identified morphologically by their large pale nuclei, bearing a prominent nucleolus, surrounded by an intensely stained cytoplasm. Glial cells (astrocytes, oligodendrocytes and microglia) were identified by their smaller nuclei and little cytoplasm and were not included in cell counts. Nissl-stained slides were quantitatively analyzed under a Nikon Eclipse 600 light microscope, using a motorized stage interfaced with the computer. The density of surviving neurons at one day from kainate injection, expressed as cells/mm, was obtained at 40x, counting nucleoli of the neurons by the use of the Neurolucida software program for computer-aided microscopy (Microbrightfield Inc., Williston, VT). Cells were counted on the computer screen using an Optronics MicroFire digital camera mounted on a Nikon Eclipse E600.

### Immunohistochemistry

Immunofluorescence staining for GFAP, P-c-Jun, neuron-specific nuclear protein (NeuN), PTEN, cleaved caspase-3 and MAP-2 was performed in the oriens, radiatum and pyramidale stratum of the CA3 hippocampus area 24 h after kainate stimulation. After blocking non-specific binding sites for 30 min at room temperature with 0.3% Triton X-100 and 10% normal donkey serum (Sigma-Aldrich) in phosphate buffered saline (pH 7.4), the sections were incubated with the primary antibodies in the same solution at 4°C: 1∶80 polyclonal rabbit anti P-c-Jun (Abcam, Cambridge, UK) together with 1∶200 monoclonal mouse anti NeuN (Millipore); 1∶500 mouse anti GFAP antibody (Cell Signaling, Plymouth, PA, USA); 1∶200 mouse anti PTEN antibody (Santa Cruz); 1∶300 polyclonal rabbit anti cleaved caspase-3 antibody (Cell Signaling Technology Inc., Danvers, MA) together with 1∶200 monoclonal mouse anti Microtubule-Associated Protein 2 (MAP-2, Millipore).

The polyclonal rabbit anti P-c-Jun antibody (Abcam, Cambridge, UK, Cat No. ab30620) was obtained by immunizing a rabbit with a synthetic phospho-peptide derived from human c-Jun around the phosphorylation site of serine 73. This antibody detects c-Jun only when phosphorylated at serine 73 (manufacturer's information obtained in human breast carcinoma and extracts of UV-treated Hela cells).

The monoclonal anti NeuN antibody (Millipore, Cat No. MAB377, clone A60) specifically recognizes the DNA-binding, neuron-specific protein NeuN.

The monoclonal mouse anti GFAP antibody (Cell Signaling, Plymouth, PA, USA, Cat No. 3670) detects endogenous levels of total GFAP protein and was obtained by immunizing animals with native GFAP purified from pig spinal cord.

The monoclonal mouse anti PTEN antibody (Santa Cruz Biotechnology, Cat No. sc-7974, clone A2B1) was raised against amino acids 388-400 of PTEN of human origin.

The polyclonal anti cleaved caspase-3 (Asp175) is produced by immunizing animals with a synthetic peptide corresponding to amino-terminals residues adjacent to (Asp175) in human caspase-3 (Cell Signaling, Cat No. 9661).

The monoclonal mouse anti MAP-2 antibody (Millipore, Cat No. MAB 3418, clone AP20) is produced by immunizing animals with bovine brain microtubule protein.

After rinsing, primary antibodies were detected by incubating sections for one hour at room temperature in 1∶200 Cy3-coniugated donkey anti-rabbit IgG (H+L) (Jackson Immuno Research Laboratories, West Grove, PA, USA) or in 1∶100 Cy2-coniugated donkey anti-mouse IgG (H+L) (Jackson Immuno Research Laboratories). Sections were counterstained by incubation with DAPI (4,6-Diamidino-2-phenyindole, dilactate; Sigma) diluted 1∶10 in PBS 0.3% Triton X-100 for 10 minutes at room temperature after immunohistochemical labelling. Controls included sections treated with secondary antibody alone, which did not show appreciable staining. The percentage of the total area in the CA3 layers that was GFAP or P-c-Jun-positive was quantified using the Image j NIH software for Windows (Bethesda, MA, USA).

### Western blot analysis

Proteins in total homogenates and in cellular fractions (mitochondria and cytosol) were determined using the Bio-Rad protein assay kit. Total cell proteins or proteins of the distinct subcellular fractions were separated on pre-cast mini-gel system with 4–12% Bis-Tris gradient (NuPAGE electrophoresis system from Invitrogen) with MES running buffer. Separated proteins were transferred electrophoretically onto nitrocellulose membranes. Equal protein loading was confirmed by Ponceau S staining. After the blocking of nonspecific binding sites with Tris-buffered saline-Tween (t-TBS; Tris, 0.02 M, NaCl, 0.150 M and Tween 20, 0.1%) containing 1% non-fat dried milk, nitrocellulose membranes were incubated with specific primary antibodies and the immune complexes were detected using appropriate peroxidase conjugated secondary antibodies and enhanced chemiluminescent detection reagent ECL (Pierce). The blots were stripped and used for sequential incubation with control antibodies. Densitometric analysis was carried out on the Western blots using the Quantity one software. The pixel intensity for each region was analysed, the background was subtracted and the protein expressions were normalized to loading control for each lane.

From total cell proteins the phosphorylation status of Akt has been evaluated incubating the nitrocellulose membrane with primary antibodies against p-Akt (1∶200 in milk, Santa Cruz) and Akt (1∶200 in milk, Santa Cruz) in order to calculate the ratio p-Akt/Akt. The immune complexes were detected using as the secondary antibody the Stabilized Goat Anti-Rabbit HRP-Conjugated by Pierce. Anti-Mouse GAPDH (1∶1000, Millipore) was used as control of loading.

Blots from mitochondrial and cytosolic fraction were incubated with anti-mouse PTEN (1∶500 in milk, Santa Cruz). The immune complexes were detected using as the secondary antibody the Stabilized Goat Anti-Mouse HRP-Conjugated by Pierce (1∶1000 in milk). As loading control it has been used anti-mouse GAPDH for cytosolic fraction (1∶1000 in milk, Millipore) and anti-rabbit HADHA for mitochondrial fraction (1∶5000 in milk, Abcam).

The polyclonal anti p-Akt antibody (Santa Cruz Biotechnology, Cat No. sc-7985-R) was raised in rabbit against a short amino acid sequence containing phosphorylated Ser 473 of Akt1 of human origin.

The polyclonal anti Akt antibody (Santa Cruz Biotechnology, clone H-136, Cat No. sc-8312) was raised in rabbit against amino acids 345-480 of Akt1 of human origin.

These antibodies can recognize bands at 56 and 62 kDa.

The monoclonal anti-PTEN antibody (Santa Cruz Biotechnology, clone A2B1 Cat No. sc-7974) was raised in mice against amino acids 388–400 of PTEN of human origin and recognizes a single band of 55 kDa.

The monoclonal anti Glyceraldehyde-3-phosphate dehydrogenase (GAPDH) antibody (Millipore, clone 6C5, Cat No. MAB374) was obtained by immunizing a mouse with Glyceraldehyde-3-phosphate dehydrogenase from rabbit muscle. This antibody recognizes a single band of 38 kDa.

The polyclonal rabbit anti HADHA antibody (Abcam, Cat No. ab54477) was obtained by immunizing a rabbit with a synthetic peptide derived from human surrounding amino acid 750. This antibody detects a single band of approximately 83 kDa.

### Statistics

Statistical analysis was performed with GraphPad PRISM 5. Values shown represent the mean ± SEM of at least three separate experiments. Data were compared with One-way or Two-ways ANOVA followed by Tukey's test or Bonferroni post hoc test. Differences were considered to be significant when P<0.05.

## Results

### 1. Epiletic seizures

In this study we studied kainate (KA) acid-treated animals that showed seizures corresponding to the fifth degree of the Racine scale. The few animals that did not reach this degree of the scale were excluded from the study. Systemic administration of KA resulted in the death of approximately 15% of the animals (data not shown). As expected, the systemic inhibitor administration does not affect animal behavior (data not shown). By Western Blotting analysis we have shown that PTEN inhibitor is able to increase the level of phosphorylated Akt, the major downstream target of PTEN, in the hippocampus 6 hours after kainate stimulation compared to the administration of kainate alone. These data confirmed that the bpv(pic) dose is appropriate and inhibits PTEN activity in this model (S1 Figure).

### 2. Morphological changes in the CA3 area in the hippocampus

**Figure 1:**
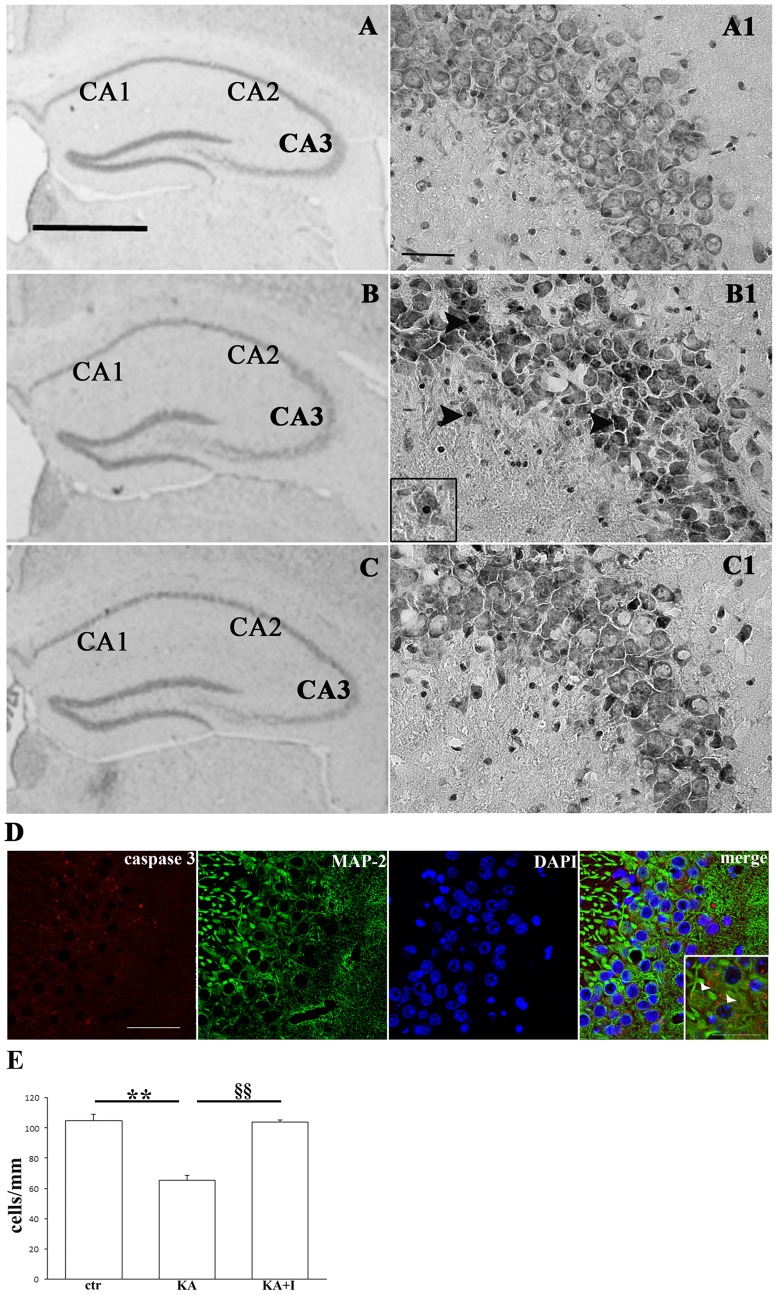
PTEN inhibition protects CA3 neurons from kainate-induced excitotoxicity. Nissl-staining of hippocampus sections of mice treated with saline (A, control), with kainate (B, KA) or with both kainate and bpv(pic) (C, KA+I), killed one day after treatment (scale bar: 1 mm). A1. CA3 area of control animals (ctr) treated with saline only (scale bar: 50 µm). B1. CA3 area of animals treated with kainate (KA). Arrowheads point to apoptotic bodies and condensed nuclei as a consequence of kainate treatment. At higher magnification, a neuron that is degenerating. C1. CA3 area of animals treated with both kainate and PTEN inhibitor (KA+I). D. Immunostaining showing cleaved caspase-3 (in red) in neurons labeled with MAP-2 (in green) in the CA3 area of the hippocampus, one day after kainate administration. At higher magnification, arrowheads indicating neurons with a clear expression of cleaved caspase-3. Scale bar: 50 µm (30 µm at higher magnification). E. Surviving neurons in the hippocampal CA3 region. Histogram showing the linear density (cells/mm) of surviving neurons in the CA3 area of the hippocampus one day following kainic (KA) acid injection or kainic (KA) acid injection and PTEN inhibition (I) by bpv(pic) (KA+I). **P<0.01, §§*P*<0.01.

Since it has been reported that systemic injection of KA resulted in a selective hippocampal neurodegeneration, especially in the CA3 area [27] for the highest density of KA receptors, we focused our analysis on this area. One day after KA stimulation, the thickness of the pyramidal cell layer in the CA3 was markedly decreased (Fig. 1B) compared to the control (Fig. 1A) whereas the application of PTEN inhibitor partially prevented the thinning of CA3 (Fig. 1C). Besides, as a consequence of the treatment with kainate, we also observed degenerating neurons with condensed nuclei that can be considered signs of apoptotic cell death in the CA3 area (see Fig. 1B1) as demonstrated by double immunofluorescence for MAP-2 (Microtubule-associated protein 2), a neuronal marker, and cleaved caspase-3, a marker of apoptosis, clearly expressed in neurons in the CA3 area after kainate treatment (Fig. 1D).

### 3. Quantitative analysis of hippocampal cell loss in the CA3 area

Subsequently, to determine the susceptibility of the hippocampal CA3 subfield to KA stimulus, we counted neurons in this area in Nissl-stained coronal sections. These sections represent three different levels of the hippocampus, from rostral to the caudal, corresponding to plates 41 (Bregma 1.22 mm), 44 (Bregma 1.58 mm) and 47 (Bregma 1.9 mm) of the Paxinos and Franklin atlas [26]. After 24 h, kainate stimulus resulted in a decrease of the pyramidal cell density, partially prevented by bpv(pic) treatment. This tendency, present at all considered levels, is statistically significant in the intermediate level (Two way ANOVA and Bonferroni post hoc analysis, P<0.01 control (ctr) vs kainate (KA); P<0.01 kainate (KA) vs kainate and PTEN inhibitor (KA+I)) (Fig. 1E).

### 4. GFAP immunofluorescence

**Figure 2:**
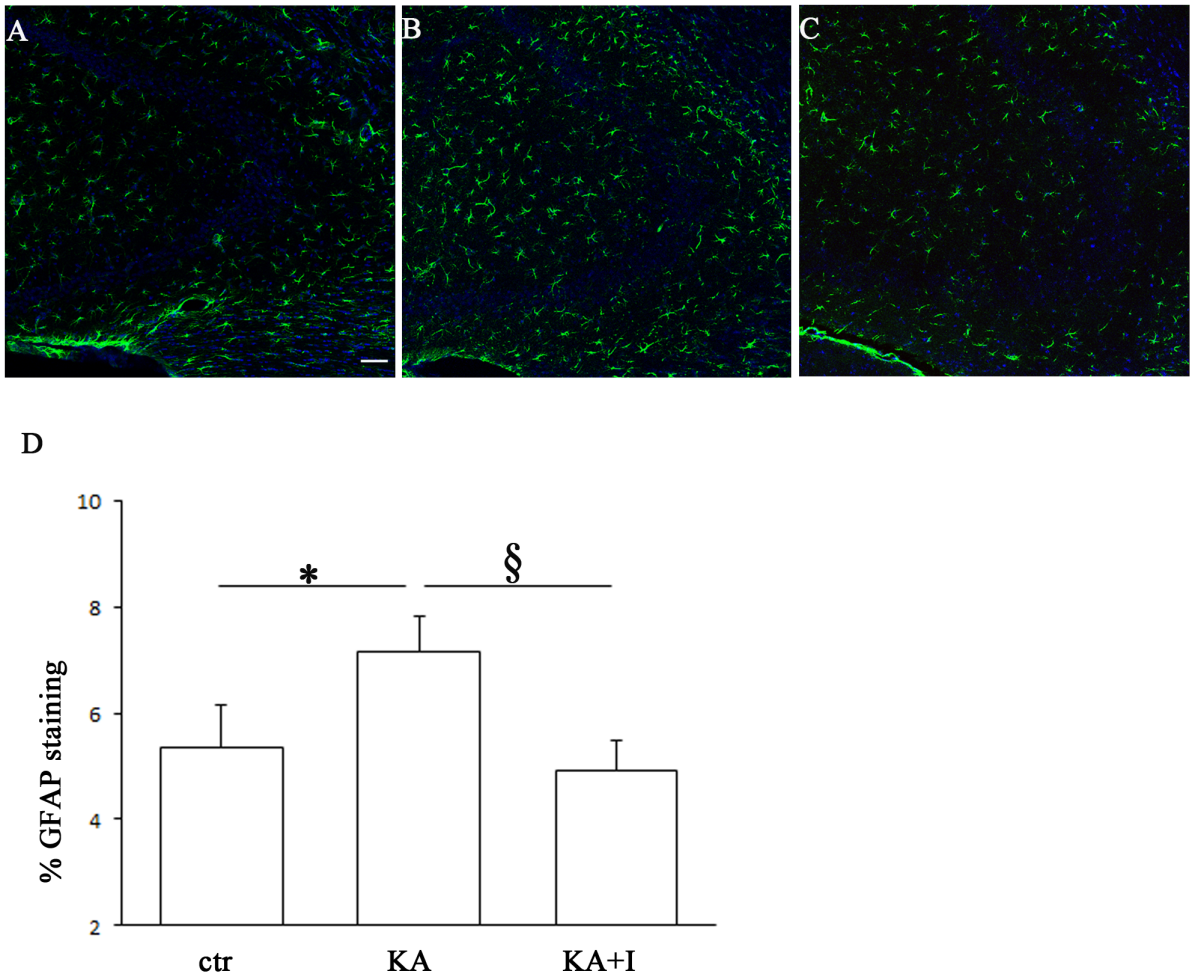
Astrogliosis in the CA3 area of the hippocampus following kainic acid injection. Glial fibrillary acidic protein (GFAP)-positive profiles in the hippocampi of control (A), kainic acid (KA)-treated, 1 day survival mice (B) and KA/bpv(pic)-treated, 1 day survival mice (C) (scale bar: 50 µm). D. Histogram showing the % of GFAP staining in the hippocampal CA3 area one day after kainic acid injection and PTEN inhibition by bpv(pic). The significant increase in GFAP immunoreactivity after kainate administration, expressed as a percentage of the whole area occupied by positive profiles, is significantly prevented by bpv(pic) treatment. *P<0.05, §<0.05.

Data in the literature showed the involvement of PTEN in the first stage of reactive astrogliosis *in vivo* [22]. To explore this issue in our experimental design, we quantified the GFAP density of immunofluorescent reaction in the stratum oriens, radiatum and pyramidale of the CA3 hippocampus area 24 h after kainate stimulation (Fig. 2A,B,C). Two-way ANOVA statistical analysis revealed that the administration of kainate as well as that of the inhibitor causes the same effects on the density of GFAP in all the 3 considered layers. Therefore we evaluated the effects of kainate on the whole CA3: 24 h after stimulus application, the density of GFAP positive profiles was significantly increased compared to the control animals which received saline (One way ANOVA and Turkey post hoc analysis, P<0.05 ctr vs KA). PTEN inhibition by bpv(pic) resulted in a significant decrease in GFAP fluorescence density compared to kainate, almost the same density as in the control (One way ANOVA and Turkey post hoc analysis, P<0.05 KA vs KA+I; P = ns ctr vs KA+I) (Fig. 2D).

### 5. P-c-Jun immunofluorescence

**Figure 3:**
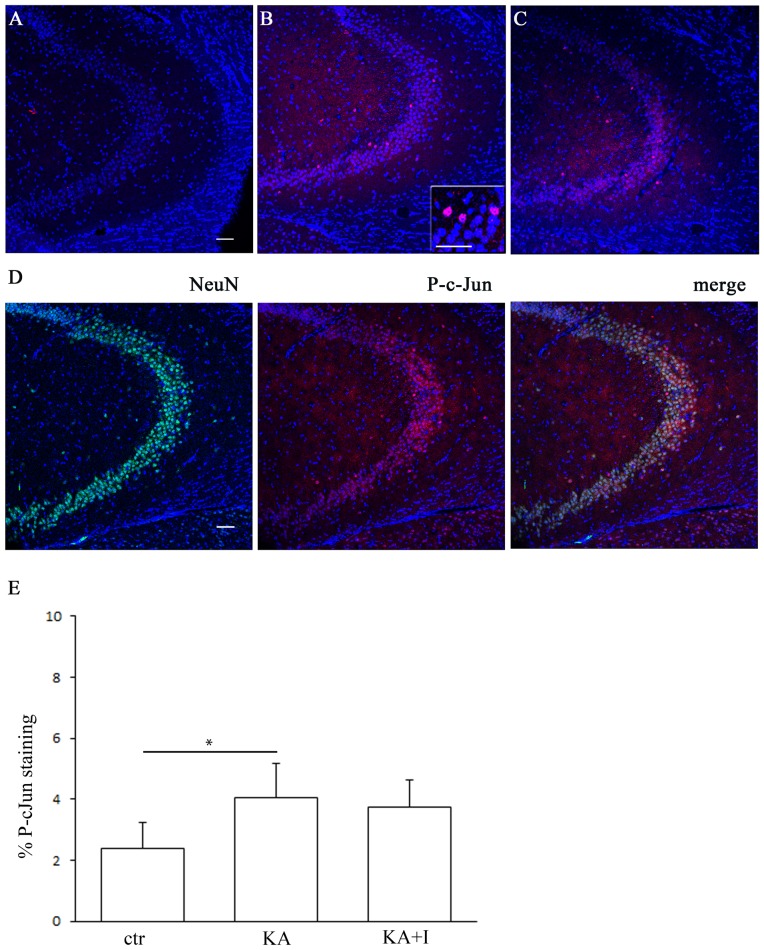
Activation of c-Jun after kainate stimulation in the CA3 area of the hippocampus. The P-c-Jun immunoreactivity, absent in control mice (A), can be observed in neurons of animals treated with kainate (B) or with both kainate and PTEN inhibitor (C), sacrificed one day after the treatment. At higher magnification, nuclei of neurons positive for P-c-Jun after kainate treatment. D. Immunostaining showing that P-c-Jun (red) is revealed in the nucleus of neurons labeled with NeuN (green) in the CA3 area of the hippocampus, one day after the kainate stimulation. Scale bar: 50 µm. E. Histogram showing the % of P-c-Jun staining in hippocampal CA3 subfield one day following KA injection and PTEN inhibition by bpv(pic). As it can be observed, bpv(pic) is unable to prevent the significant increase in the P-c-Jun immunoreactivity caused by kainate treatment. *P<0.05.

Zhang and co-workers [15] revealed the presence of a link between PTEN and JNK signaling pathways, in a model of *in vivo* ischemic damage. According to this finding, we hypothesized that PTEN inhibition could affect the phosphorylation/activation status of c-Jun, a downstream nuclear target in the JNK pathway. As for GFAP, we measured the P-c-Jun density in immunofluorescent reaction in the nuclei of neurons in the stratum oriens, radiatum and pyramidale of the CA3, 24 h after kainate stimulation (Fig. 3A,B,C).

Also in this case, Two-way ANOVA statistical analysis showed that the administration of kainate as well as that of the inhibitor causes the same effects on the density of P-c-Jun immunofluorescence in all the 3 considered layers. Therefore we considered the effects of kainate on the whole CA3: after 24 h from stimulus application, the fluorescence density of P-c-Jun was significantly increased compared to the control animals treated with saline (One way ANOVA and Turkey post hoc analysis, P<0.05 ctr vs KA) (Fig. 3E). Double immunofluorescence for P-c-Jun and for neuron-specific nuclear protein (NeuN), a neuronal marker, revealed a colocalization of the two markers in the same cells, indicating that P-c-Jun immunoreactivity was localized in neurons as shown in Fig. 3D. Surprisingly PTEN inhibition by bpv(pic) did not result in a decrease in the fluorescence density of P-c-Jun compared to the kainate (One way ANOVA and Turkey post hoc analysis, P = ns KA vs KA+I; P = ns ctr vs KA+I) (Fig. 3E).

### 6. PTEN immunofluorescence

**Figure 4:**
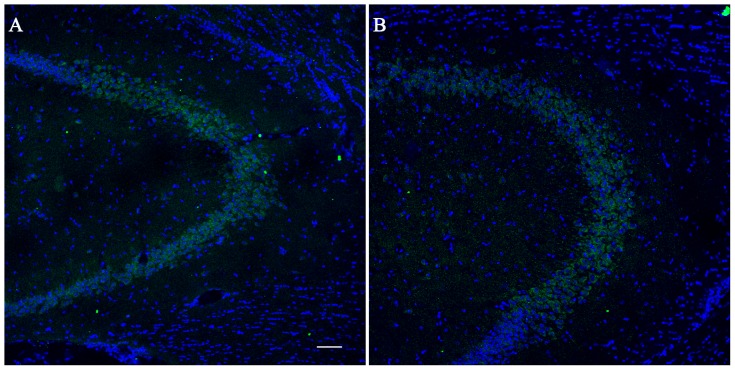
PTEN expression in the CA3 area of the hippocampus. Immunofluorescence showing PTEN expression in the CA3 area 1 day after kainic acid i.p. injection (B) and in control mice treated only by saline (A). Nuclei were stained with nuclear marker DAPI. Scale bar: 50 µm.

PTEN-positive profiles were found in CA3 of the hippocampus in the saline controls (Fig. 4A) and one day after kainate stimulation (Fig 4B).

### 7. Mitochondrial translocation of PTEN after the excitotoxic stimulus

As already mentioned, a death stimulus may also result in a subcellular redistribution of PTEN compared to the physiological status. For example, in cultured hippocampal cells stimulated by staurosporine, PTEN displayed a mitochondrial accumulation [13]. By Western Blotting analysis we evaluated if a mitochondrial translocation of PTEN also occurred in our model of *in vivo* kainate excitotoxicity.

**Figure 5:**
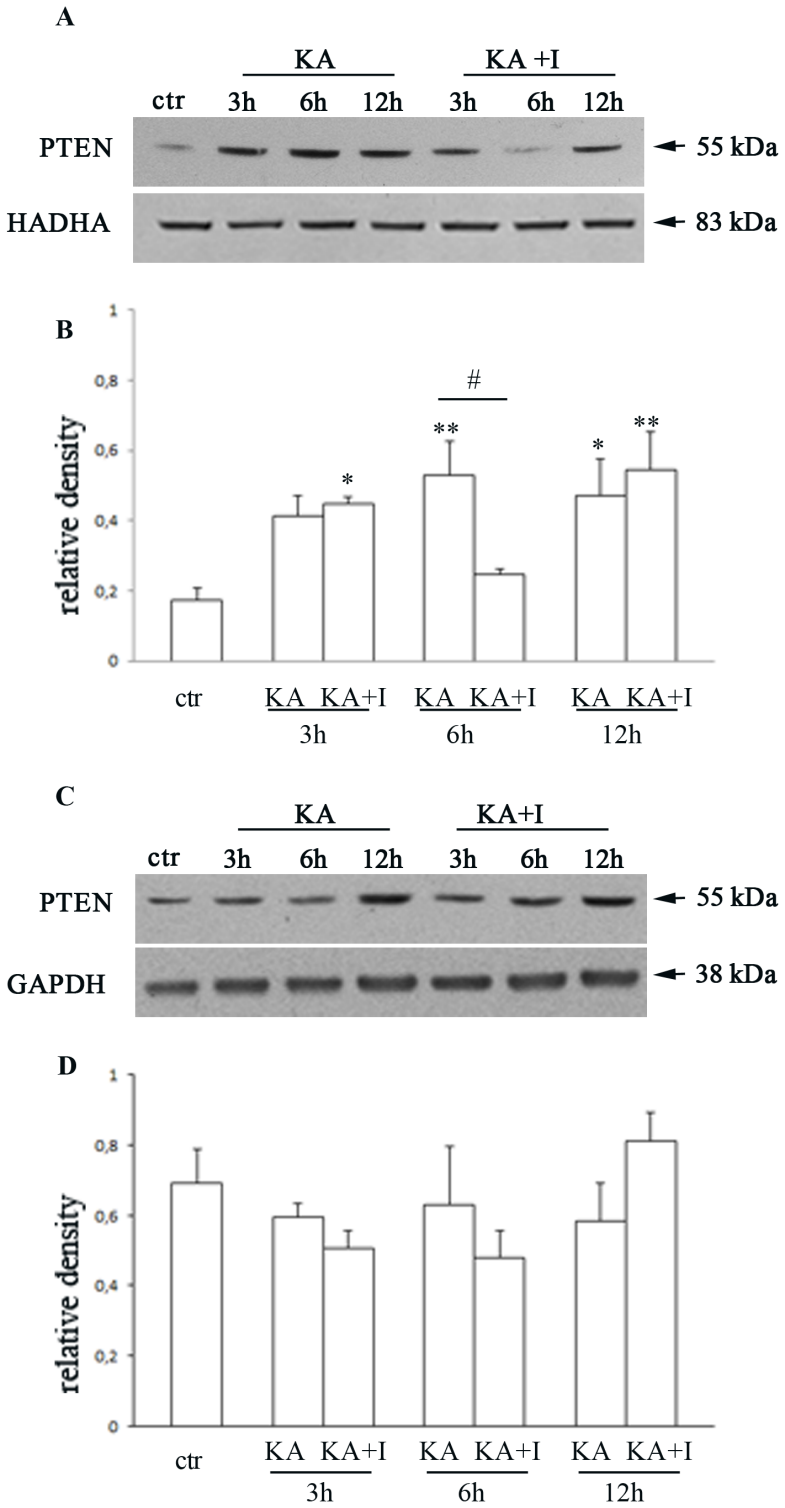
PTEN expression in mitochondrial and cytosolic fraction after the excitotoxic stimulus. Representative Western blot (A) and relative quantification (B) showing mitochondrial PTEN translocation in control (ctr), kainic treated group (KA) and kainic/bpv(pic) group (KA+I) 3, 6 and 12 h after the kainate treatment. As it can be observed, the excitotoxic stimulus leads to a significant increase in mitochondrial PTEN expression, significantly decreased 6 h after kainic acid injection in animals treated with bpv(pic). On the contrary, in the cytosolic fraction kainic acid or PTEN inhibitor does not lead to significant differences in PTEN levels in any time points considered as it can be observed from representative Western blots (C) and relative quantification (D). Data are expressed as mean ± S.E.M., *P<0.05, #*P*<0.05, **P<0.01 with Two way ANOVA and Bonferroni post hoc test. Experiments were repeated three times.

We evaluated the mitochondrial PTEN 3, 6 and 12 h after kainate stimulation: a significant increase in mitochondrial accumulation of PTEN occurred 6 and 12 h after kainate systemic administration compared to the control animals treated with saline (Two way ANOVA and Bonferroni post hoc test P<0.01 ctr vs KA 6 h; P<0.05 ctr vs KA 12 h). A significant decrease in mitochondrial PTEN levels seems to be 6 h after the application of the excitotoxic stimulus in animals treated with PTEN inhibitor (Two way ANOVA and Bonferroni post hoc test P<0.05 KA 6 h vs KA+I 6 h) but this effect was no longer visible 12 h after the application of kainate (Two way ANOVA and Bonferroni post hoc test P = ns KA 12 h vs KA+I 12 h) (Fig. 5A-B).

On the contrary, in the cytosolic fraction, PTEN levels did not show significant differences after kainate stimulation or PTEN inhibition in any time point considered (Fig. 5C-D).

## Discussion

Although excitotoxicity is considered as the major mechanism regulating neuronal death in many human disease states, an effective neuroprotective strategy against excitotoxicity is still far. The most common neuroprotective strategy consisting in blocking excitatory aminoacid NMDA receptors, although it proved neuroprotective, leads also to the inhibition of pathways necessary for normal neuronal function and survival, generating many adverse side effects [28], [29], [30], [31]. These findings suggest the necessity to intervene at an intermediate level in the excitotoxic cascade, targeting molecules downstream of the calcium entry but upstream the level where it is impossible to block neuronal death, in order to prevent the inhibition of physiological functions.

To this aim the phosphatase and tensin homolog deleted on chromosome 10 (PTEN), initially known as tumor suppressor factor, can be considered as an ideal candidate. Several studies proved its key role in regulating neuronal death and the neuroprotective value of its downregulation/inactivation: neurons from PTEN heterozygous mice exhibited increased resistance to death induced by overactivation of glutamate receptors [12]; pretreatment with bpv(pic), a potent PTEN inhibitor or by intra-cerebroventricular infusion of PTEN siRNA reduced the infarct volume in a male rat model of middle cerebral artery occlusion [6]; PTEN downregulation by a specific antisense oligonucleotide or by siRNA significantly inhibited the elevated production of reactive oxygen species, a key event in neurodegenerative processes, and the neuronal death in *in vitro* models of stroke and Parkinson's disease [32]; recently, elevated levels of PTEN mRNA and protein have been shown in human Alzheimer's Disease (AD) where they contribute to neurodegeneration [14].

Traditionally it is thought that PTEN regulates neuronal death through its lipid phosphatase activity, directly antagonizing the prosurvival PI3K/AKT signaling pathway [10], [11] although, recently, additional PTEN dependent mechanisms as the poor characterized PTEN phosphatase activity [9], different PTEN subcellular localizations [13], [33], [34] and the link with excitotoxic signaling pathways, such as c-Jun N-terminal kinase cascade, are emerging [15]. However, despite the several studies on the role of PTEN in neuronal death, its complex mechanisms have not yet been completely clarified.

The aim of this work was to study the role of PTEN in excitotoxic damage in a model of murine temporal lobe epilepsy obtained by intraperitoneal injection of kainic acid. Systemic or intracerebral injection of kainate results in sustained epileptic activity in the hippocampus, similar to human temporal lobe epilepsy with an important neuronal loss in the hilus, in the CA1 and in the CA3 area of the hippocampus [35] and with the activation of microglia and astrocytes in the hippocampal lesions [36], [37].

The involvement of PTEN in the kainate excitotoxic damage is proved inhibiting PTEN with two intraperitoneal injections of bisperoxovanadate bpV(pic), 30 minutes before and 90 minutes after stimulation with kainate. We observed that one day after kainate stimulation, the thickness of the pyramidal cell layer in the CA3 area was markedly decreased compared to the control, in agreement with the significant reduction in pyramidal neuronal density resulting from the stereological counts at three different levels of the hippocampus in the same area. As expected, PTEN inhibition by bpv(pic) resulted in a rescue of the pyramidal neuronal loss in the CA3 area in all the tree considered hippocampus levels.

Challenging with kainate leads also to astrogliosis, the activation process of astrocytes characterized by hypertrophy and by the upregulation of intermediate filaments composed of nestin, vimentin, and glial fibrillary protein (GFAP) and by the activation of cell proliferation [38], [39]. The reactive astrocytes migrate towards the injured area to constitute the glial scar, and release factors mediating the tissue inflammatory response and remodeling after lesion [40], [41] although when neuronal damage is irreversible, microglia and astrocytes start secreting toxins rather than trophic factors, thus exploiting neuronal damage [42], [43]. The presence of kainate receptors on astrocytes leads to their activation thirty minutes after kainate injection [36], [37]; at the same time PTEN appears weakly expressed in immature astrocytes. Decreased PTEN expression in highly activated astrocytes showing extensively spindled shape seven days after kainic acid injection, suggested that PTEN could have a role mainly in the early stage of reactive astrogliosis in vivo [22]. As expected, treatment with kainic acid has considerably increased the presence of mature astrocytes whereas the application of PTEN inhibitor, resulting in a decrease in the levels of GFAP expression compared to the mice treated only with kainate, seems to be opposed to the process of astrocytes maturation/activation. Therefore, using a reactive gliosis model by systemic injection of kainic acid in mice we confirmed that PTEN has a role in the early stage of reactive astrogliosis *in vivo* and with further analysis we could explain what this entails in terms of neuroprotection or neurodegeneration. In fact, providing all this evidence, we have only underlined the crucial role of PTEN in excitotoxicity once again without investigating the molecular mechanisms in which it is involved.

As known, the c-Jun N-terminal kinases (JNKs), belonging to the family of mitogen-activated protein kinases (MAP-kinases), are key enzymes in the cellular response to stress signals such as NMDA stimulation, Abeta fragments, hypoxia, reactive oxygen species, ultraviolet radiations. Data from our lab showed the involvement of JNK in excitotoxic neuronal death induced by intraperitoneal injection of kainate in adult male Sprague-Dawley rats [44]. Other studies underlined the link between JNK signaling pathway and PTEN so that the activation of c-Jun N-terminal kinases 1/2 (JNK 1/2) is decreased after the specific downregulation of PTEN protein level and is associated to increased AKT activation in ischemic damage [15]. This evidence suggested us to verify the effects of PTEN inhibition on the phosphorylation/activation status of c-Jun, the downstream target in JNK signaling cascade, evaluating the fluorescence density of P-c-Jun in the neuronal nuclei of the CA3 area of mouse hippocampus. As expected, treatment with kainate increased significantly P-c-Jun immunoreactivity in the nuclei of the pyramidal neurons although the application of PTEN inhibitor was not able to decrease the fluorescence density of P-c-Jun. Therefore we hypothesize that PTEN and JNK signaling pathways, although both involved in the kainate excitotoxicity, act independently from each other and PTEN regulates the neuronal death through molecular mechanisms that do not include c-Jun as a downstream target.

Through the proposed mechanisms of action, the different subcellular localizations of PTEN assumed an increasing importance. In particular the mitochondrial localization of PTEN in cultured hippocampal cells after challenging with staurosporine seems to contribute to mitochondria-dependent apoptosis as the knockdown of PTEN inhibited the staurosporine-induced increase of cytochrome c release in the cytosol and the activation of caspase 3, key features of apoptotic process [13]. Besides Bononi and co-workers showed that a fraction of PTEN protein localizes to the endoplasmic reticulum and mitochondria-associated membranes, signaling domains involved in the transfer of calcium from the endoplasmic reticulum to the mitochondria and in the apoptosis induction [45]. These pieces of evidence and the knowledge that in our model recurrent seizures associated with the chronic phase of epilepsy lead to the mitochondrial damage [46] suggested us to verify PTEN mitochondrial accumulation after kainate stimulation. We performed Western Blotting analysis 3, 6 and 12 h after kainate stimulation revealing a mitochondrial accumulation of PTEN that seems to be significantly reduced 6 hours after the excitotoxic stimulus in animals treated with bpv(pic). This effect is no longer visible after 12 h, probably due to the reversible binding between PTEN and its inhibitor.

To explain the presence of PTEN in mitochondria, it has been suggested a role for PTEN as a mitochondrial carrier of Bax, a proapoptotic protein localized at the mitochondrial membrane upon apoptosis induction. It has been proved that Bax is physically associated with PTEN in the absence and in the presence of the staurosporine apoptotic stimulus so as to suggest a role for PTEN in mitochondrial damage and, consequently, in mitochondrial apoptosis [13].

In our model the fundamental role ascribed to PTEN in the massive neuronal loss in the CA3 area of the hippocampus seems to be associated to the PTEN mitochondrial translocation significantly reduced 6 h after kainate application in animals treated with bpv(pic). Further analysis, such as the analysis of the mitochondrial apoptotic pathway, will provide the evidence to support this hypothesized role of PTEN in mitochondrial apoptosis.

The PTEN pathway plays a key role in excitotoxic cell death in our experimental model of epileptic seizures in the hippocampus. Its blockade can prevent both neuronal cell death and glial activation. The pathway is probably independent of the JNK pathway. Therefore, they could act either in concert or in alternative, but an efficient neuroprotective strategy should target several different pathways of cell death to be more efficient in the translation from bench to bedside.

## Supporting Information

S1 Figure **Increase of p-Akt level by bpv(pic) administration.** Histogram (B) and images (A) showing Akt activation in protein total extracts of the hippocampus 6 hours after kainate treatment. As it can be observed, PTEN inhibition by bpv(pic) antagonizes kainate effect on Akt activation, increasing the level of p-Akt, although not significantly (One way ANOVA and Turkey post hoc analysis, P = ns KA vs KA+I; P = ns ctr vs KA; p = ns ctr vs KA+I).
(TIF)
