# Supplementary figures and images for: Neuroprotective and Anti-Inflammatory Roles of the Phosphatase and Tensin Homolog Deleted on Chromosome Ten (PTEN) Inhibition in a Mouse Model of Temporal Lobe Epilepsy
Source: PLoS One. 2014 Dec 12;9(12):e114554. doi: 10.1371/journal.pone.0114554 (PMC4264755; doi:10.1371/journal.pone.0114554)

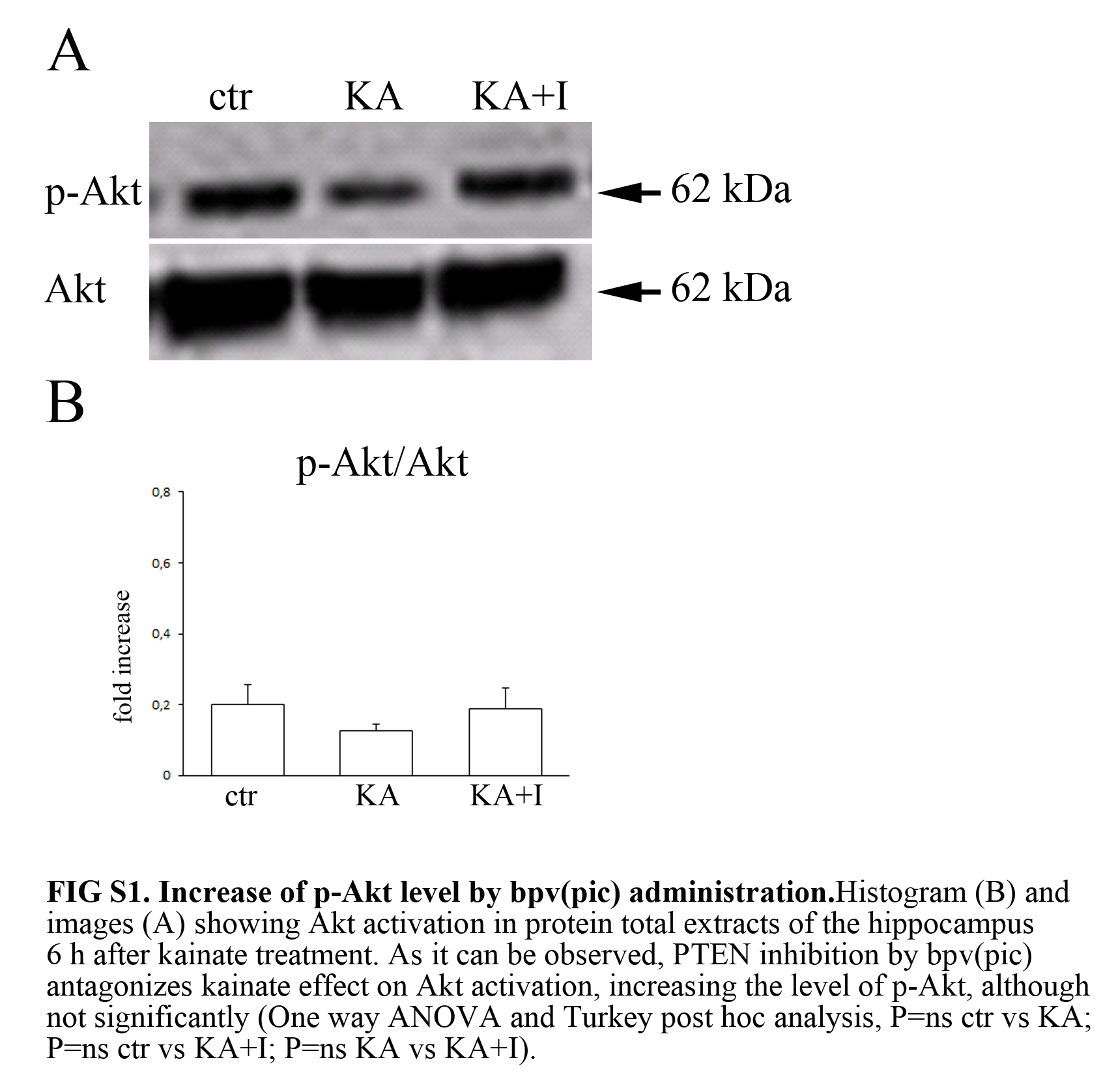

Supplement: S1 Figure — Increase of p-Akt level by bpv(pic) administration. Histogram (B) and images (A) showing Akt activation in protein total extracts of the hippocampus 6 hours after kainate treatment. As it can be observed, PTEN inhibition by bpv(pic) antagonizes kainate effect on Akt activation, increasing the level of p-Akt, although not significantly (One way ANOVA and Turkey post hoc analysis, P = ns KA vs KA+I; P = ns ctr vs KA; p = ns ctr vs KA+I). (TIF) [file pone.0114554.s001.tif]
